# Respect for the journey: a survivor-led investigation of undergoing psychotherapy assessment

**DOI:** 10.1007/s00127-020-02017-1

**Published:** 2021-01-31

**Authors:** Alison Faulkner, Katie Kelly, Sarah Gibson, Steve Gillard, Lana Samuels, Angela Sweeney

**Affiliations:** 1 Independent Researcher, London, UK; 2 Tower Hamlets Early Intervention for Psychosis Service (THEIS), East London NHS Foundation Trust, London, E2 6BF UK; 3 Independent Researcher, Kent, England; 4 School of Health Sciences, City, University of London, London, EC1V 0HB UK; 5 PEER, St George’s, University of London, Cranmer Terrace, London, SW17 0RE UK; 6 Service User Research Enterprise, King’s College London, London, SE5 8AF UK

**Keywords:** Psychotherapy assessments, Trauma, Survivor, Client experience, Qualitative, Survivor research

## Abstract

**Purpose:**

Psychotherapy assessments are key decision points for both clients and services, carrying considerable weight on both sides. Limited research indicates that assessments have immediate and long-term impacts on clients, particularly where trauma has been experienced, affecting engagement with therapy. Understanding assessments from clients’ perspectives can inform service development and improve client experience.

**Methods:**

This is a survivor-led exploration of clients’ experiences of undergoing assessment for talking therapies. Interviews were conducted with seven people who had undergone assessment for psychological therapies in third sector and NHS services. Interviews were recorded, transcribed and analysed thematically.

**Results:**

The core theme was ‘respect for the journey’ reflecting the need expressed by participants for their life experiences prior to the assessment to be given full respect and consideration. Six sub-themes were identified: trauma and desperation, fear of judgement, search for trust and safety, sharing and withholding (a balancing act), feeling deconstructed, and finding hope.

**Conclusions:**

The findings highlight the heightened emotional power surrounding psychotherapy assessments, reflecting the journey participants had undertaken to reach this point. The dilemma facing clients at the heart of an assessment—how much to share and how much to withhold—demonstrates the importance for services and assessors of treating the journey a client has made to the assessment with care and respect. Findings indicate the value of services and practitioners undertaking a trauma-informed approach to assessment encounters.

**Supplementary Information:**

The online version contains supplementary material available at 10.1007/s00127-020-02017-1.

## Introduction

In England, many people undergo talking therapy assessments every year, including approximately one million people in the state-funded Improving Access to Psychological Therapies (IAPT) programme alone [1]. Many who seek psychotherapy (an umbrella term we are using for a broad range of talk-based therapies including cognitive behaviour therapy and counselling) are likely to have experienced significant trauma (we adopt a broad definition of trauma to include, for instance, witnessed or experienced interpersonal and community violence, childhood maltreatment and social and historical traumas [2]); it is estimated that around half of all women with mental health problems have experienced some form of abuse [3, 4].

Psychotherapy assessments are key decision points, opening or closing individual opportunities and enabling service providers to rationalise service offers in the context of limited resources [5]. Assessment procedures are typically bespoke to service providers and modalities, varying from unstructured history gathering to the use of structured assessment tools. Most offer a rare opportunity for people to tell their story, a potentially therapeutic act in itself [4]. Within mental health, assessment protocols are often pre-determined by technical frameworks, with little space for personal meaning making [6]. Reinterpretation of experience through the prism of illness and diagnosis can render it almost impossible for individual story-telling and meaning-making. A similar process may occur in psychotherapy assessments whereby the prism of the modality comes to serve as the technical framework through which people’s stories are reinterpreted and (mis)understood.

Research has found that rigid psychotherapy assessments, including form-filling, can create difficult experiences for service users [7], complicating access to therapy and contributing to drop-out [8]. While research into women’s experiences of feminist/women’s therapy found that assessments can be cathartic and healing, with good outcomes sometimes traceable to assessments [9], this often contrasts with previous experiences of pathologisation [9, 10]. Similarly, research into male sexual abuse survivors’ therapy experiences found that not being asked about abuse contributed to people feeling that assessors were not listening [7].

A systematic review of adults’ experiences of psychotherapy assessments [11] found that assessments had a significant impact on clients, including the potential to open up trauma without sufficient support and having long waits for therapy, despite significant distress and crisis, sometimes resulting in drop-out [12]. Similarly, Kantor found that trauma survivors are often reluctant to enter psychotherapy for fear of re-experiencing their trauma through telling their story [13]. The review identified collaboration and therapeutic alliance as factors that had the greatest positive impact on assessment experiences. Whilst assessments have both immediate and long-term impacts on clients and their engagement with therapy, and trauma survivors sometimes report poor experiences [7, 9, 10], there is little research specifically exploring trauma survivors’ experiences of psychotherapy assessments, and the systematic review identified a lack of independent service user research in the field. The current study aims to fill these research gaps.

In this survivor-led study, we report the lived experience of undergoing psychotherapy assessments, particularly where there are past, recent or current experiences of trauma.

## Methods

Our study is survivor-led: the majority of the research team have direct experiential knowledge of the topic of enquiry [14, 15] and our knowledge and values “guide[s] the whole research process—from formulating the research questions to drawing conclusions” [14]. Thus, rather than attempting to generate knowledge through positivist principles of distance, objectivity and neutrality [16], our approach is located within the interpretive/qualitative paradigm which acknowledges the role of our subjectivity in attempting to explore the lived experiences of participants, with reflexivity used to explore the role and impact of this subjectivity [17]. Our study was guided by a Service User (SUAG) and a Clinician (CAG) Advisory Group. SUAG members were diverse in terms of sexual orientation and ethnicity, though dominated by cis women. SUAG members contributed to all aspects of the study, with the detailed study design guided by a service user Ethics Working Group (AF, KK, SG and LS), drawn from the SUAG. Ethics approval was granted by Camberwell and St Giles Research Ethics Committee (18/LO/0077).

### Sampling and recruitment

Participants were recruited from psychotherapy services in a large metropolitan area in England representing different therapy modalities: two-third sector services (for adults abused in childhood and a Women’s Centre), two National Health Service (NHS) Improving Access to Psychological Therapy services (including one for people with Severe Mental Illness), and one NHS tertiary (i.e. highly specialised) trauma service. Services were selected where clients were socio-demographically diverse, including high proportions of people from Black and Minority Ethnic backgrounds. Table 1 provides information on participants and their assessment.

**Table 1:** Participant demographics and assessment processes

Setting	Participant demographics	Assessment process
IAPT	Female, White British, 20–29, heterosexual	Face-to-face assessment, waiting list place, second face-to-face assessment, therapy beginning shortly afterwards
IAPT	Male, White British, 30–39, heterosexual	Telephone assessment, gap, second telephone assessment, waiting list place, then one face-to-face assessment with the therapist (first therapy session)
Community	Male, Chinese, 40–49, heterosexual	One face-to-face assessment with therapy beginning the following week
Community	Female, White British, 30–39, heterosexual	One face-to-face assessment then a waiting list place. Interview conducted after therapy had been completed
Community	Female, White British, 30–39, heterosexual	One face-to-face assessment then a waiting list place. Interview conducted after therapy had been completed
NHS	Female, White British, 40–49, heterosexual	Three face-to-face assessment sessions then an outcome meeting
NHS	Female, Black British African*,* 30–39, heterosexual	Ten face-to-face assessments, then referral for preliminary treatment elsewhere

Assessors were recruited through researcher contact with services; assessors then identified and approached eligible clients. Inclusion criteria for clients were people aged 18+ who had undergone or were about to undergo assessment. Assessors were encouraged to approach clients who identified as belonging to minoritized groups.

### Interviews

Interviews were conducted in 2018 by a survivor researcher (AS) in a setting chosen by participants. Semi-structured interview schedules were developed through reflecting on our experiential knowledge, informed by narrative [2] and systematic reviews [11]. Key areas of enquiry included why assessments were sought and experiences of trauma enquiries. We used a visual timeline as a data elicitation tool to record the assessment process (see online material). All participants gave written informed consent.

Care was taken to assure participants they did not need to reveal anything they did not wish to. Consequently, participants revealed different amounts of information about their past experiences and the reasons they were seeking therapy.

### Analysis

Interviews were audio-recorded, transcribed and anonymised. Transcripts were analysed thematically [18] by survivor researchers, reflexively drawing upon our shared personal experiences of undergoing psychotherapy assessments to inform the interpretative process [17, 19, 20]. This is consistent with reflexive thematic analysis which asserts that, “qualitative research is about meaning and meaning-making, and viewing these as always context-bound, positioned and situated” [17]. Thus, our aim was to produce an explicitly survivor-generated analytic account.

Three analysts (AF, KK and AS) read the first three transcripts, identifying early ideas and themes. Following discussion, AS then generated an early coding frame which was applied to the data using MAXqda Plus 2018 (version 18.0.8).

Once data were collected and coded, AF reviewed the emerging analysis, synthesising codes with explanatory power in capturing the essence of lived assessment experiences. This generated a second coding frame which was discussed reflexively with AF and AS, then re-applied to the raw data by AF. Finally, AF developed a written account of the lived experience of assessments from the coded data, in discussion with AS.

The emerging analysis was discussed in a data workshop with Advisory Groups following the approach of Shimmin et al. [21]. We also explored connections between our psychotherapy assessment experiences and how these related to gender, ethnicity, socioeconomics and sexual orientation [21].

## Results

Two men and five women participated. Five participants identified as White British; one woman identified as Black British African and one man identified as Chinese. Two participants were recruited through a National Health Service trauma service, a Women’s Centre, and IAPT services. One was recruited through a specialist charity. Most participants were interviewed between 4 days and 4 weeks after their assessment. However, in one service, we were obliged to interview women after they had completed therapy, meaning that their assessments were between one and three years prior to the interview.

Rich discussions with the Advisory Group led to substantial validation of the core themes, particularly ‘respect for the journey’ and refinement of some sub-themes, such as the ‘balancing act’ between withholding and sharing at the heart of the negotiation taking place within assessments.

### Findings

Through the survivor-led analytic process, we saw participants’ experiences as a journey leading to the assessment and its aftermath. Their journey might begin with difficult experiences in childhood or early adulthood, continue through recent and current life events, leading to a decision to seek help. The assessment could then present a formidable hurdle or potential opportunity. Consequently, the assessment becomes the present-day focal point for the emotional weight people have been carrying, giving rise to the build-up of complex and difficult feelings.

We describe this journey in three parts: the emotional weight of the journey to the assessment; the assessment itself and the need to find respect for the journey; and the aftermath, with its potential for hope alongside a sense of exposure and deconstruction.

#### Trauma and desperation

Most participants described past experiences of trauma and abuse, revealing a complex history of childhood abuse or neglect and experiences in adulthood, creating a build-up of feelings and emotions that overwhelmed their capacity to cope in the present.

“and…because with my case there’s several things; it wasn’t one you know incident led to this, it was several things and um…with er, partly um, ... emotional abuse or emotional neglect—or actually he just called it ‘neglect in childhood’. [in-breath] And all this has just cooked up this big mess.” Participant 2

The effects of past trauma often made it hard for people to cope with daily life: flashbacks and memories, irritability and temper, panic, hearing voices, hallucinations, lack of sleep and drinking too much and/or eating too little.

Feelings of shame and self-blame associated with past abuse and trauma, had prevented some people from seeking help for many years. The feeling of shame was most commonly associated with past sexual abuse or domestic violence.

“So you feel this shame, over the years I’ve just been holding things, suppressing, suppressing, and that’s why I now have to deal with this thing in a bigger way because half the time it’s been suppressed, or you are not heard properly and given the right treatment.” Participant 4

Several participants talked graphically of a period of desperation before seeking help. One woman described losing ‘my marbles and my body and everything’ (participant 4), as she progressively lost weight and lost touch with hers. One man used the powerful metaphor of walking around with a prosthetic leg to describe this period of gradually realising that he was, in effect, psychologically disabled and needed to take action.

“[...] and I’ve had this prosthetic leg for 20-odd years um, wearing clothes no-one notices that I’ve got a prosthetic leg because I know how to use it, I know…it just, it’s just part of me and everyone recognises me as a person with two legs. [...], and the pretence of being normal or having…you know, all four fully-functional limbs is becoming harder and harder.” Participant 1

A common difficulty was the effect of trauma and distress on people’s relationships with friends and family, and particularly children, a factor that became a strong motivation for seeking help. One participant was worried about making ‘the same kind of mistakes that my parents did’ and that his relationship with his child might deteriorate if he did not seek help. Another participant described the pressure to appear ‘normal’ in front of her children.

“My kids are at school. I don’t want to be letting them see this vulnerable side all the time. I wanted them to come back home to a normal home, so just before they got back, I felt like I was putting on this front and it was killing me because I wasn’t necessarily being myself, but I didn’t want them to suffer anymore.” Participant 4

The trauma in someone’s past was often brought to the surface by a life event or crisis in the present, such as violence from a partner, illness, bereavement or the age of a child. This could mean that present life became disrupted by memories, overwhelming feelings and flashbacks.

“You know I’ve been dragging this shit round since I was [in-breath] a teenager; I can’t…I’ll be {age} in January, I don’t wanna keep dragging it around and round. It’s had enough and I want it over.” Participant 2

#### Fear of judgment

For most people, the assessment became the present-day focal point for the desperation and accumulated trauma of a lifetime. This placed an enormous weight of anxiety and anticipation onto the assessment: to reveal something deeply personal and sometimes shaming, to risk being judged and yet to prove themselves worthy of help. This weight of anticipation was exacerbated for those who had already waited years to seek help. The prospect of talking to a stranger about traumatic experiences and feelings was daunting, particularly for those revealing things for the first time. Some spoke of their expectations or hopes for change. One woman described wanting to find a space where she could finally be honest about her experiences and feelings:

“I think I was really looking for a platform where I could be really honest. [In-breath] Um, I tried the speaking to people at … church … but you can’t always be like completely honest. Um, talking to family you can’t be honest because feelings are hurt. [...] I just needed to just be messy and I, I don’t feel actually there’s any other way you can be messy.” Participant 3

The predominant sentiment expressed about the assessment was the fear of being judged, scrutinised, potentially found wanting or unworthy of therapy. It was likened to facing an exam, a job interview or work appraisal, in which judgment would be inherent. The sense that the assessment had the potential to provide hope and help or rejection, gave it considerable significance and power.

“this assessment… feels like it’s either hope or it’s the end. Absolutely. It is. It’s gonna make or break you; it’s, it’s such a big deal by the time you get there.” Participant 2

Two participants described how past experiences of trauma and abuse eroded their ability to trust other people, particularly in the context of assessment or judgment. They expressed concern that people with abuse histories could be re-traumatised by the lengthy assessment processes and the potential for being judged, and might never get to an assessment:

“It’s difficult enough as it is to even come out to talk to somebody about it and then having to do all these other things it seems... if you don’t have the courage or personality or tenaciousness to go through these things then you will never be able to get any help. And, like, I’m quite sure quite a lot of people don’t.” Participant 1

#### The assessment: respect for the journey

The strongest theme to emerge from the assessment itself was the need to have the emotional weight brought to the assessment, from sometimes extended journeys, respected and given space.

“And when you eventually do get in front of somebody, as long as respect is paid of the journey that person has been through […] I think as long as they understand that it’s safe.” Participant 2

Respect was characterised by a sense of the assessor coming alongside the person in direct contrast to their fear of someone sitting in judgment over them. People talked of appreciating authenticity, compassion, the assessor being ‘human’ and showing ‘genuine concern’ or making ‘human connection’ with them.

“So, for me personally I felt that’s the first time I’ve seen somebody who I thought ‘you are actually listening to me and you can identify that I need further assistance’. Something that I’d had to live with because no one was really picking it up and being able to help me.” Participant 4

#### The search for trust and safety

Many interviewees expressed a desperate need to be able to trust and to feel safe, intensified by the accompanying fears, shame and self-doubt that made safety and trust so hard to achieve.

“I just felt really comfortable. I felt this, it was like a gut instinct that I knew that I could trust this woman. I knew that there was no judgment, that I could talk to her openly about everything that had happened.” Participant 5

A significant building block for establishing trust was the assessor’s authentic validation of traumatic experiences, whether simply believing them to naming something that had previously remained unnamed. This had the power to contradict the fear of judgment described in the build-up to the assessment. Compassionate validation of people's experiences re-framed feelings and behaviours as understandable responses to trauma, enabling people to begin feeling believed and worthy of therapy.

“She would say to me ‘no you are not crazy it is part of the impact of what you are going through’. And that started making me feel a little bit more normal because I started isolating myself because I was scared how people would judge me. To be told that this is normal, this is acceptable, normal and acceptable in the sense of what you are going through made sense.” Participant 4

The wider service could also engender trust, for instance where the environment conveyed a ‘homely’ feel:

“if I went there and it was like a really sterile kind of like clinical environment that wouldn't kind of work." Participant 1

For others, it was sharing clear and transparent information and demonstrating reliability: being ‘true to their word’ (P4). Even a long wait for therapy could be ameliorated by an assessor being ‘painfully honest’ about resources: ‘there’s nothing worse than not knowing what's happening’ (P2). Some assessors were praised for explaining the entire assessment process and the nature of therapy.

Many people referred to assessors’ personal qualities and skills as helping them to feel fully heard and safe enough to disclose: that they were friendly, approachable and yet professional. They also appreciated their expertise; for example, their trauma knowledge.

Most women preferred to speak to, and have therapy with, a woman, particularly if they had experienced violence and abuse. Being in a women’s centre could create a feeling of refuge:

“So, yes, I certainly wouldn’t have been able to open up if it was a man. Just it being a woman and in a [women's service] made me feel completely safe, like completely safe.” Participant 5

#### Sharing and withholding: a balancing act

For most participants, a complex dynamic surrounded the sharing or withholding of experiences and feelings. Some were acutely aware that sharing too much might leave them open and exposed, and potentially feeling ‘wretched’.

“But also I didn’t wanna sit and just fall apart, I didn’t wanna start crying, I didn’t wanna sit and [in-breath]…because then once that happens you’ve gone and you’re so vulnerable; it’s like you’re just open; it’s like someone’s just cracked your chest open and you’re open and vulnerable.” Participant 2

Some assessors made their intention to share control of the assessment explicit, enabling people to have some choice over the information they shared and assessment’s pace. They encouraged people to withhold and keep themselves safe, reassuring them that revealing everything was not necessary.

“[Assessor name] did explain that. You don’t have to share everything if it’s too much for you because she said about she didn’t want me to leave and be in a bad place from saying everything. So, that was reassuring.” Participant 7

Nevertheless, several participants remained uncertain about how much to reveal, leading to a complex balancing act.

“...the one thing that stopped me being really open was not really knowing what was expected of me. [...] you know, it’s a trust issue isn’t it—you have to build up the trust that they’re not gonna judge you.” Participant 3

#### The aftermath: exposure and hope

In the assessment’s aftermath, feelings of relief and hope were expressed, the after-effects of disclosure, and some anxiety and apprehension about the start of therapy. Some were told they had a long wait for therapy which caused anxiety about how they would manage in the interim. A few had developed strategies to cope with the effects of trauma, or had strategies recommended to them by the assessor.

#### Feeling deconstructed

Although some participants had been careful not to expose too much of themselves, feelings and symptoms in the assessment’s aftermath could still be challenging, like ‘someone’s deconstructing you’ (P2). One woman described the sense of something unfinished in the session and the impact of this on her journey home:

“...by the time I’ve finished I’m thinking, yes, I’m OK and then I walk out and then I get my flashbacks and whatever and to be honest when I was leaving to sit on public transport to go was the biggest fear. I wish I could fly home.” Participant 4

#### Finding hope

Some participants talked of feeling hopeful, either during, or because of, the assessment. They were hopeful that they would receive therapy, and that they could recover. Several people talked about the value of self-care strategies, either in the immediate aftermath of assessments or whilst waiting for therapy. Several assessors suggested strategies based on trauma-informed approaches and some participants were referred to other services for interim support.

“So I felt quite positive that I was already trying to put things in practice, so I thought I’m going to benefit from this rather than me just talking about whatever is going on in my life that distracts me from going on to try and actually deal with the actual stuff, the inner stuff.” Participant 7

## Discussion

Our analysis found that the essence of clients’ psychotherapy assessment experiences is the need to feel that their journey to the assessment is respected, recognizing the emotional weight the assessment holds [22]. This can be understood in the context of the damaging effects of trauma alongside the need and wait for therapy. We situate our findings in the context of trauma-informed approaches which can help to mitigate these effects [23–25]. ‘Respect for the journey’ is partly an expression of the profound ambivalence often felt about opening up and sharing as against withholding and ‘heeding the natural tendency to protect oneself due to the implicit vulnerability that opening oneself up to others entails’ [page 89 [26]]. It also reflects the weight of expectation brought to the assessment, characterised by the strength of emotions described and the need to feel safe to trust the assessor with their stories, their lives. Many people do not disclose their experiences of trauma for years [27]; focusing this on one opportunity puts enormous pressure on the individual and the assessment. In a survivor-led study, Morris found that, whilst some women opened up, for others, it was important to establish a therapeutic relationship first [9]. This speaks to the balancing act surrounding judging how much to reveal and the risks inherent in revealing too much, particularly if the assessment might lead nowhere. Revealing too little also carries a risk of failing to justify the need for therapy.

A key principle of trauma-informed approaches is the need for sensitive trauma enquiries: asking questions in respectful, sensitive, timely and appropriate ways; offering a choice regarding whether or not to answer; and understanding the potential for re-traumatisation caused by describing traumatic events [2]. Ferentz argues that trauma-informed psychotherapy assessments should unfold carefully as a process, rather than as one-off events, with disclosures responded to compassionately and with a strong focus on containment and safety [23].

Nevertheless, the value in finding a safe space in which to tell one's story also comes through in this study, a theme that resonates throughout survivor literature and Mad Studies [28, 29]. Nehls and Sellman [4] identified the therapeutic impact of simply telling one’s story for women with abuse histories, substance use and mental health problems. Morgan et al. also describe the value of personal narratives in helping us ‘make sense of our lives, our identities and our worlds’ [page 82 [6]].

People with trauma histories described a powerful fear of judgment. Experiences of trauma and abuse are linked to feelings of shame, humiliation, embarrassment, and dread [30]: feelings that can be hard to voice. This is particularly profound where people, often women, have experienced ‘betrayal trauma’ where the abuse has been perpetrated by someone close to them, who they relied upon for survival [31].

Whilst this is a small study, with no claims to transferability to large diverse populations, small samples are typical of in-depth qualitative explorations [7]. Rather than achieving transferability, our aim was to explore the lived experience of undergoing assessment, particularly for trauma survivors. Doing so within a survivor-led approach meant that experiential knowledge was elevated to the central way of knowing about and making sense of the world, generating a rich, grounded account of undergoing assessment. In keeping with the ethos of survivor research, we have emphasised hearing the voices of participants through the lengthy results section [32]. Whilst the reliability of this account was increased through the lengthy analytic process involving multiple analysts, the knowledge generated is inevitably partial and situated. However, this partial, situated knowledge—based firmly on survivor perspectives—can be considered a strength. Gillard et al. [33], for instance, found that service user analysts produce accounts that differ from mainstream researchers, focusing for instance on experiences and emotions, rather than procedures and processes. Similarly, our situated account explores the emotional weight of assessments and the impact this has on trauma survivors. Finally, recruiting participants via their assessors may have resulted in selection bias, although knowing that interviewers had some shared experience can enable greater openness in interviewees [33].

### Implications and further research

Trauma-informed approaches (TIAs) prioritise the prevention of re-traumatisation and iatrogenic harm that can occur through service encounters [2]. Our findings suggest there is the potential for re-traumatisation within assessments, not only through telling one’s story in a context where help might not be offered, but also due to the weight of anticipation brought to the assessment and the potential for non-empathic responses. Trauma-informed approaches help make sense of the finding that clients need to feel safe and to trust the assessor before they can disclose past trauma. Our study suggests that achieving this might involve: prioritising honesty and transparency; clearly communicating a non-judgmental approach; engaging collaboratively; ensuring warmth, compassion and authentic listening; normalising and validating responses to violence and abuse; and providing strategies to help people manage the wait for therapy because of the potential damage of long waits and their impact on assessment encounters.

Researchers are increasingly concerned that the experiences of survivors are at the heart of what they do. Our study demonstrates that survivor-led research can generate rich understandings of lived experiences. Collaboration was key to generating our analytic account, with team members occupying dual roles across categories of researcher/survivor/assessee [34, 35]. Future research into experiences of violence and abuse could prioritize elevating the experiential through survivor-led and collaborative approaches, ensuring that lived experiences are central.

Given the predominance of White British women in the study, further research is needed, including with, for instance, men, older adults, people with disabilities, people who identify as Black and Minority Ethnic, and people on the LGBTQIA spectrum.

## Conclusion

Prospective therapy clients, with and without trauma experiences, are asking for their journey to the assessment, and the story they bring, to be treated with respect and without judgment. This study highlights the heightened emotional power surrounding psychotherapy assessments, particularly for people with experiences of violence and abuse, and the corresponding importance of trauma-informed approaches. The dilemma at the heart of an assessment—how much to share and how much to withhold—is one that clients face with considerable apprehension. It is imperative that services and assessors treat this with respect through efforts to enable people to feel safe and begin building trust.

### Supplementary Information

Below is the link to the electronic supplementary material.

Supplementary file1 (DOCX 63 KB)

Supplementary file2 (DOCX 61 KB)

## Data Availability

The datasets generated and analysed during the current study are not currently publicly available as participants did not give informed consent for this.
