# Correction to: Respect for the journey: a survivor-led investigation of undergoing psychotherapy assessment

**DOI:** 10.1007/s00127-021-02053-5

**Published:** 2021-03-01

**Authors:** Alison Faulkner, Katie Kelly, Sarah Gibson, Steve Gillard, Lana Samuels, Angela Sweeney

**Affiliations:** 1 Independent Researcher, London, UK; 2 Tower Hamlets Early Intervention for Psychosis Service (THEIS), East London NHS Foundation Trust, London, E2 6BF UK; 3 Independent Researcher, Kent, England; 4 School of Health Sciences, City, University of London, London, EC1V 0HB UK; 5 PEER, St George’s, University of London, Cranmer Terrace, London, SW17 0RE UK; 6 Service User Research Enterprise, King’s College London, London, SE5 8AF UK

## Correction to: Social Psychiatry and Psychiatric Epidemiology 10.1007/s00127-020-02017-1

The original version of this article contained references to articles and authors that were blinded. These should read as follows:

Semi-structured interview schedules were developed through reflecting on our experiential knowledge, informed by narrative (Sweeney et al. 2016) and systematic reviews (Sweeney et al. 2019).

SUAG members contributed to all aspects of the study, with the detailed study design guided by a service user Ethics Working Group (authors 1, 2, 3 & 5).

Interviews were conducted in 2018 by a survivor researcher (author 6).

The original article has been corrected.

