# Supplementary material for: Respect for the journey: a survivor-led investigation of undergoing psychotherapy assessment
Source: Soc Psychiatry Psychiatr Epidemiol. 2021 Jan 31;58(12):1803–11. doi: 10.1007/s00127-020-02017-1 (PMC10628034; doi:10.1007/s00127-020-02017-1)
Supplement: Supplementary file 1 — Supplementary file1 (DOCX 63 KB) [file 127_2020_2017_MOESM1_ESM.docx]

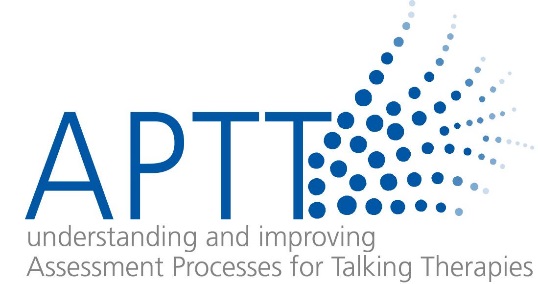


# Respect for the journey: a survivor-led investigation of undergoing psychotherapy assessment - Supplementary Material 1

SERVICE USER TIMELINE OF THE ASSESSMENT PROCESS

First contact

Please add:

- Steps in the assessment process and approximate dates / waiting times
- Interim contacts with, or support from, the service
- When informed of the outcome of the assessment
- When therapy started or is anticipated to start
