# Supplementary material for: Respect for the journey: a survivor-led investigation of undergoing psychotherapy assessment
Source: Soc Psychiatry Psychiatr Epidemiol. 2021 Jan 31;58(12):1803–11. doi: 10.1007/s00127-020-02017-1 (PMC10628034; doi:10.1007/s00127-020-02017-1)
Supplement: Supplementary file 2 — Supplementary file2 (DOCX 61 KB) [file 127_2020_2017_MOESM2_ESM.docx]

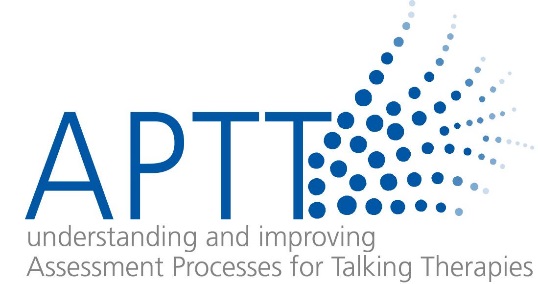


# Respect for the journey: a survivor-led investigation of undergoing psychotherapy assessment - Supplementary material 2

SERVICE USER INTERVIEW GUIDE

*Opening:* complete the timeline

*Topic 1:* Can you tell me a little bit about why you were assessed for a talking therapy?

*Topic 2:* Can you describe what happened in the assessment?

*Topic 3:* Can you tell me a little bit about what your assessor was like?

*Topic 4:* Did you want to be assessed by someone of a particular gender, ethnic background, sexual orientation or language?

*Topic 5:* Did you feel you had the information you needed through the assessment process?

*Topic 6:* Can you describe whether you felt involved in decisions about your support?

*Topic 7:* Trauma

We are asking the people we interview whether they have experienced trauma, and whether this was discussed in the assessment. We are not asking about people's specific experiences of trauma in this research. We understand that talking about trauma can be uncomfortable or distressing and you are free to choose not to answer any of my questions or to take a break or stop the interview. Shall I go ahead or would you like to skip to the next question?

- Can you describe your experiences of being asked about trauma?

*Topic 8:* How did you feel immediately after your assessment?

In the few days after your assessment?

Now?

*Topic 9:* Have you been informed yet of the outcome of your assessment?

*Closing:* Recommendations and closing thoughts
